# Efficient Bayesian inference for stochastic agent-based models

**DOI:** 10.1371/journal.pcbi.1009508

**Published:** 2022-10-05

**Authors:** Andreas Christ Sølvsten Jørgensen, Atiyo Ghosh, Marc Sturrock, Vahid Shahrezaei

**Affiliations:** 1 Department of Mathematics, Faculty of Natural Sciences, Imperial College London, London, United Kingdom; 2 PASQAL SAS, Massy, France; 3 Department of Physiology and Medical Physics, Royal College of Surgeons in Ireland, Dublin, Ireland; University of California Riverside, UNITED STATES

## Abstract

The modelling of many real-world problems relies on computationally heavy simulations of randomly interacting individuals or agents. However, the values of the parameters that underlie the interactions between agents are typically poorly known, and hence they need to be inferred from macroscopic observations of the system. Since statistical inference rests on repeated simulations to sample the parameter space, the high computational expense of these simulations can become a stumbling block. In this paper, we compare two ways to mitigate this issue in a Bayesian setting through the use of machine learning methods: One approach is to construct lightweight surrogate models to substitute the simulations used in inference. Alternatively, one might altogether circumvent the need for Bayesian sampling schemes and directly estimate the posterior distribution. We focus on stochastic simulations that track autonomous agents and present two case studies: tumour growths and the spread of infectious diseases. We demonstrate that good accuracy in inference can be achieved with a relatively small number of simulations, making our machine learning approaches orders of magnitude faster than classical simulation-based methods that rely on sampling the parameter space. However, we find that while some methods generally produce more robust results than others, no algorithm offers a one-size-fits-all solution when attempting to infer model parameters from observations. Instead, one must choose the inference technique with the specific real-world application in mind. The stochastic nature of the considered real-world phenomena poses an additional challenge that can become insurmountable for some approaches. Overall, we find machine learning approaches that create direct inference machines to be promising for real-world applications. We present our findings as general guidelines for modelling practitioners.

## Author summary

Computer simulations play a vital role in modern science as they are commonly used to compare theory with observations. One can infer the properties of a system by comparing the data to the predicted behaviour in different scenarios. Each scenario corresponds to a simulation with slightly different settings. However, since real-world problems are highly complex, the simulations often require extensive computational resources, making direct comparisons with data challenging, if not insurmountable. It is, therefore, necessary to resort to inference methods that mitigate this issue, but it is not clear-cut what path to choose for any specific research problem. In this paper, we provide general guidelines for how to make this choice. We do so by studying examples from oncology and epidemiology and by taking advantage of machine learning. More specifically, we focus on simulations that track the behaviour of autonomous agents, such as single cells or individuals. We show that the best way forward is problem-dependent and highlight the methods that yield the most robust results across the different case studies. Rather than relying on a single inference technique, we recommend employing several methods and selecting the most reliable based on predetermined criteria.

This is a *PLOS Computational Biology* Methods paper.

## Introduction

Mathematical and computational modelling opens up and sheds light on various research questions in fields, ranging from hydrodynamics to oncology [1–4]. They have thus become crucial to advances in nearly all research areas and for addressing a variety of real-world problems. However, in many cases, detailed mechanistic mathematical models of nature are notoriously complex and computationally cumbersome. Direct statistical analyses can become insurmountable due to the associated computational cost when faced with such models. In this paper, we address ways to mitigate this drawback.

We focus on a specific subset of heavy computational simulations, known as agent-based models (ABMs) [5]. Such models keep track of autonomous agents (individuals, cells, or particles) that follow a set of rules prescribing their behaviour relevant to the particular phenomenon being modelled. Importantly, these rules are often stochastic and involve interactions among the agents and between the agents and their environment. If a simulation is repeated, its output changes in complex ways. Before one might compare the predictions of such ABMs to observations, one must gauge this stochasticity, which might consume considerable computational resources. Even without this additional complication, proper (Bayesian) inference requires a large number of simulations to ensure a robust exploration of the model’s parameter space, which could be large [6]. However, ABMs for real-world applications often require many agents or complex interactions, making simulations computationally demanding. Due to the high computational cost, a direct route for simulation-based inference, such as Approximate Bayesian computation (ABC) methods [7, 8], might thus be impractical.

There are different problem-dependent ways to deal with the computational challenges that complex inference problems pose. For example, some models lend themselves to statistical techniques that greatly reduce the number of required simulations [9]. In other cases, one might resort to interpolation in an existing archive of models or to parameterizations of emergent properties [10, 11]. In recent years, it has become increasingly popular to train machine learning (ML) methods to tackle the issue [12, 13]. We follow this latter approach here. In general, ML methods aim to capture the behaviour of the simulations across the parameter space to bypass the need for further simulations when performing the inference task.

ML schemes can be employed in two different ways. First, one can create a computationally efficient surrogate model, called an emulator, that mimics relevant aspects of the ABM, much like the aforementioned parameterizations [14, 15]. The emulator might then enter into a Bayesian framework, such as ABC, in the same way that the original simulations would. Secondly, one might directly use ML approaches for inference [13]. We will refer to this latter method as a direct inference machine. Whether one considers using emulators or direct inference machines, one is left with the choice of the specific ML algorithm. However, it is not clear-cut what the optimal choice is for any particular research question. Recent literature takes advantage of advances in density estimation by neural networks and focuses on methods that estimate the likelihood or posterior density (or ratio) [12, 13]. In this paper, we take a less explored approach based on an approximation to Bayesian neural networks [16, 17]. We compare the performance of this approach with methods based on Gaussian processes [18, 19] and a mixture density network [20, 21].

For this purpose, we consider two examples of real-world applications of ABMs: tumour development and the spread of infectious diseases. ABMs are used extensively in both fields [22–27]. The employed ABMs are complex enough to capture essential aspects of and resemble real-world problems going beyond toy models, although they are not as sophisticated as many of those models used to analyze real-world data [28, 29]. Their high level of complexity is an important feature of our models. In the literature, it is commonplace to use much simpler models when benchmarking Bayesian frameworks or machine learning methods. Benchmark models might thus include linear combinations of Gaussian posteriors or deterministic models, such as the Lotka-Volterra equations, to which noise is subsequently added [13]. Other canonical test cases include simple chemical reaction systems [30] (cf. the section *The Schlögl Model* in S1 Appendix). It is among such benchmarking efforts and Bayesian inference tasks that our work is placed.

For both our ABMs, we create synthetic data (also called mock observations) using the ABMs and infer the underlying model parameters using a broad range of inference algorithms. The synthetic data in our examples are considered experimentally accessible. This is a deliberate choice since real-world applications must always consider data availability. In this paper, we aim to infer the values of the underlying model parameters when faced with the synthetic data. By using synthetic data, we can compare the inferred parameter values with the ground truth, i.e. the parameter values used to create the synthetic data. We thus perform a self-consistent test that allows us to evaluate and validate the performance of the different inference techniques.

We present several implementations of emulation-based inference and direct inference machines. We apply these inference methods to the aforementioned ABMs and critically assess the results from each method. While we focus on these two examples, cancer and infectious diseases, we aim to provide general guidance for choosing which algorithm to use when dealing with computationally heavy simulations. We hereby aim to contribute to the ongoing discussion on how to effectively combine simulator-based models and stochastic biological systems with existing Bayesian approaches [31, 32].

## Methods and models

**Fig 1:**
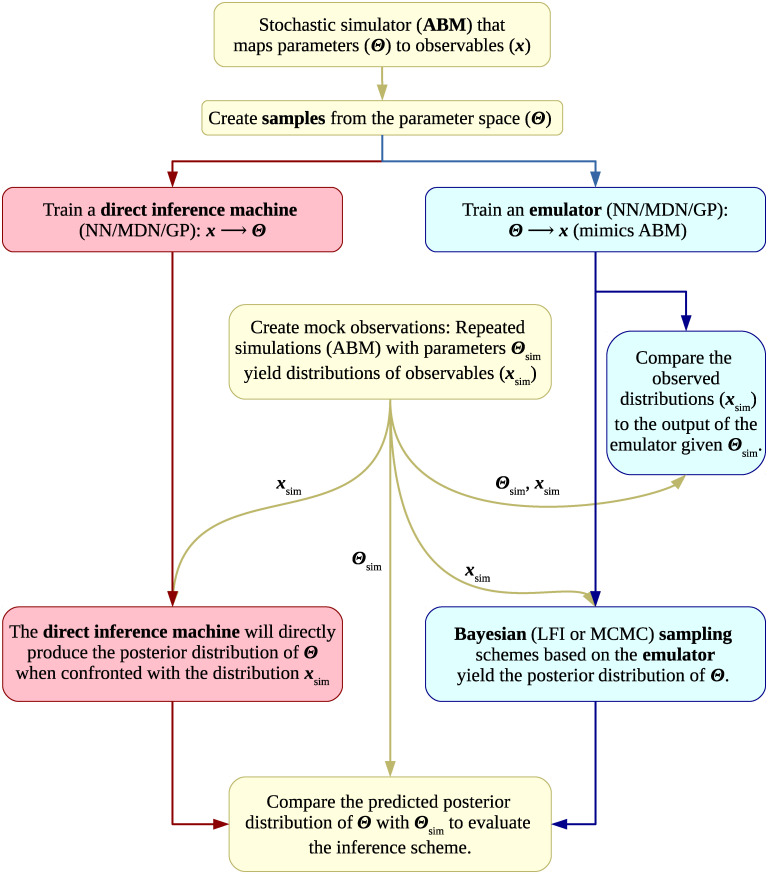
Flowchart summarizing the different steps of our analysis. We sample the parameter space by creating quasi-random grids of simulations using the agent-based models (ABM). We then employ the sampled parameter sets (**Θ_sim_**) and the simulation output (***x***_sim_) to train different machine learning (ML) methods: Neural networks (NN), mixture density networks (MDN) and Gaussian processes (GP). These ML methods can either be used as emulators or direct inference machines. Emulators mimic the simulations at low computational cost. They can be employed in Bayesian sampling schemes, such as MCMC and Likelihood Free Inference (LFI), to infer model parameters based on the observations. Direct inference machines can be seen as black boxes that produce samples from the posterior distribution of model parameters when given the observations. We use synthetic data obtained from the ABM which allows us to compare the obtained posterior distributions for the model parameters with the ground truth. For both the emulators and direct inference machines, we thus perform a classical inference task, amounting to a self-consistent test. Moreover, we quantify how well the emulators capture the behaviour of the ABMs in separate comparisons.

The flowchart in Fig 1 presents a stylised summary of the steps involved in our analysis. Below, we give a detailed overview of these steps and the employed models.

This paper presents a Bayesian analysis. We thus seek to infer the posterior probability (density), *p*(**Θ**|***x***), i.e. the probability distribution of the model parameter values (**Θ**) given the observations (***x***, cf. the section *Synthetic data*) and any prior information. In this paper, we do not impose any prior information beyond the fact that we constrain our analysis to a limited region of the respective parameter space (cf. the section *Model grid*), i.e. we impose uniform priors. Using the ABC method or the direct inference machines discussed below, we map the posterior distribution directly without consulting the likelihood, *p*(***x***|**Θ**), i.e. the probability of the observations given the model parameter values and the prior information. When using the MCMC, on the other hand, we sample from the posterior by introducing a surrogate likelihood and drawing on Bayes’ theorem:

$$\begin{array}{c} p(\Theta |x)\propto p(x|\Theta )p(\Theta ), \end{array}$$ (1)

where *p*(**Θ**) denotes the prior probability distribution, i.e. the imposed constraints on the parameters in the form of the explored region of interest.

### Agent-based models

In this paper, we use two agent-based models (ABMs) describing two distinct real-world problems: Our first model deals with a malignant type of brain cancer called glioblastoma multiforme. The second model describes the spread of infectious diseases in a population. The sections *Brain Tumour, CA model* and *Epidemic, SIRDS model* in S1 Appendix provide detailed accounts of both ABMs. Since these models are to be understood as examples that we have used to benchmark different inference methods, we limit ourselves to remark on a few critical aspects of the models here. In both models, the agents live in a two-dimensional plane, and the dynamics of the system are governed by a set of stochastic rules that dictate the behaviour of the individual agents.

We note that the stochastic processes intrinsic to the ABMs become one of the main selection criteria for choosing suitable ML methods. We illustrate this by applying the same inference tools discussed below to address a non-spatial stochastic model in the section *The Schlögl Model* in S1 Appendix.

#### Brain tumour, CA model

Our agent-based brain cancer model is a cellular automata (CA) model, in which each agent represents a tumour cell. This ABM has four input parameters. Our first two parameters, *P*_div_ and *P*_cd_, are probabilities associated with the rules for cell division and spontaneous cell death. The third parameter, λ_C_, determines the nutrient consumption of the cells and entails the possibility of cell death due to nutrient deprivation. This parameter is a rate given in units of the nutrient consumption per cell per time step.

To explain the fourth parameter, we need to mention that brain tumours are composed of three main different cell types [33]. In the following, we refer to these as glioblastoma stem-like cells (GSC), glioblastoma cells in the propagating progeny (GPP), and glioblastoma cells in the differentiating subpopulation (GDS), respectively. The fourth parameter, *ϵ*, thus denotes a probability that is associated with the differentiation of stem-like cells during cell division. All four parameters are varied on a linear scale from 0.01 to 0.50 (see S1 Appendix for further details).

In this paper, we perform inference based on synthetic data, i.e. mock observations. To do so, we need to consider what data would be available in a real-world scenario. For example, in brain cancer research, data often comprise detailed snapshots of tumour growth. To reflect this circumstance, we assume that we know the time frame during which the tumour has evolved. For the purpose of inference, we hence collect data from all simulations at a fixed snapshot (*t* = 100). To compare the model predictions to the synthetic observations, we focus on the emergent macroscopic properties of the ABM. As our summary statistics, we use five numbers describing the system as a whole: the number of GSCs, GPPs, GDSs, and dead tumour cells, as well as the total number of alive cells in the proliferating rim (cf. *Brain tumour, CA model* in S1 Appendix).

#### Epidemic, SIRDS model

In our model for the spread of infectious diseases, the agents are people. These individuals fall into four different groups as signified in the acronym of the model: SIRDS. The first group comprises people who are susceptible (*S*) to the disease. The second group includes people who are infected (*I*). In the third group, we find those people who are immune after recovering (*R*) from the disease. The last group includes those who have died (*D*) from the disease. As the infection spreads through the population, individuals move between groups.

Our SIRDS model has five input variables. The first parameter, *P*_i_, denotes the probability of contracting the disease upon contact with an infected individual. The second parameter, *P*_d_, denotes the probability of dying from the disease. The agents have fixed social relations (at home, at school, or at work). In addition, they have *N*_re_ random encounters. The logarithm of *N*_re_ constitutes our third parameter. As our fourth parameter, we choose the number of days (*t*_i_) that an infected individual is contagious. Finally, our fifth parameter is the logarithm of the length of time, log *t*_p_, during which a recovered individual is protected from getting the disease. After this time span, the previously recovered individual becomes susceptible (*S*) again.

Note that the parameters of our SIRDS model are different from one another in terms of the explored parameter range, i.e. the priors. This property stands in stark contrast to the parameters of the cancer CA model, which all share the same uniform prior, as discussed above. Also, the models are quite distinct in terms of mechanistic details (see the sections *Brain Tumour, CA model* and *Epidemic, SIRDS model* in S1 Appendix). Thus, by construction, the cancer CA and SIRDS models complement each other.

Regarding the summary statistics, we again resort to the system’s emergent properties. Rather than relying on a single detailed snapshot, epidemiologists often have comprehensive knowledge of *D*(*t*), *I*(*t*), and *R*(*t*). Here, we boil these time series down to five numbers: the total duration of the epidemic, the total death toll, the highest number of infected individuals during the outbreak, the time at which the peak in infections occurs, and the highest number of recovered agents during the outbreak (cf. the section *Parameters and summary statistics* in S1 Appendix).

We stress that both our cancer CA and our epidemiological SIRDS simulations are rather simplistic. However, they are sufficiently realistic to capture the main aspects of the real-world applications, including the stochastic nature of the modelled events. This feature is the most essential for our purposes. Rather than providing detailed models for specific biological systems, we investigate how to infer properties from observations based on such simulations. By keeping the models simple, we lower the computational cost making the analysis more practical and allowing us to readily explore different aspects.

#### Model grid

To perform inference, we construct a set of grids in the input parameter spaces with a limited number of simulations. We then use these to train the machine learning (ML) techniques discussed in the sections *Emulators* and *Direct inference* below.

We impose uniform priors on all parameters. Thus, our parameter spaces are four and five-dimensional hypercubes for the cancer CA and epidemiological SIRDS model, respectively. We could cover the parameter spaces by sampling points in regular intervals. However, this approach is rather inefficient. Alternatively, we could generate points randomly, but this approach leads to unintended clustering. We, therefore, follow the suggestion by Bellinger et al. [34] and generate points based on a Sobol sequence [35]. In doing so, we sample the hyperspace uniformly while minimizing redundant information as discussed by Bellinger et al. [34].

Table 1 lists the lower and upper boundaries of our priors, i.e. the boundaries of the sampled parameter space.

**Table 1:** Upper and lower boundaries for the model parameters (Θ) in the constructed grids.

Parameter	Lower limit	Upper limit
*P*_div_	0.01	0.50
*P*_cd_	0.01	0.50
λ_C_	0.01	0.50
*ϵ*	0.01	0.50
*P*_d_	0.00	1.00
*P*_i_	0.00	1.00
log_10_(*N*_re_)	-2.0	2.0
*t*_i_ [days]	1.0	30.0
log_10_(*t*_p_) [days]	-2.0	4.0

For both ABMs, we construct four grids with different resolutions. These four grids contain 10^2^, 10^3^, 10^4^, and 10^5^ simulations, respectively. Each simulation has a unique parameter set, i.e. the grids do not contain repetitions. Rather, the ML algorithms must infer the stochasticity of the simulations based on the variation between neighbouring simulations across the grid.

Since we cover a relatively broad region of each parameter space, we run into scenarios where all tumour cells die before *t* = 100 or where the disease is not passed on. As further discussed in the section *Synthetic data*, these scenarios are of no biological interest since they would not yield conclusive real-world data and hence not be studied. They are, at least for our purpose, effectively noise for the ML algorithms. By excluding them from the training sets, we boost the performance of the various inference techniques. More specifically, in connection with the cancer CA model of brain tumours, we remove all simulations for which the number of GSCs or GPPs is zero. As regards our epidemiological SIRDS model, we remove all cases in which the disease did not spread beyond the originally infected individuals, or the disease was not eradicated after 1825 days (5 years). The latter exclusion criterion stems from the simulation set-up (cf. S1 Appendix). For both ABMs, these criteria reduce the original grid sizes by roughly 10 per cent.

#### Synthetic data

We construct a synthetic dataset to discuss the performance of the different inference techniques. For each ABM, we generate 250 new input parameter sets and repeat each simulation 250 times to sample the resulting distributions of the output variables. These distributions can be compared to the predictions of the emulators discussed in the section *Emulators* while the original input parameters (**Θ**_*o*_) of the simulations can be compared to the posterior distributions obtained by inference. We present the metrics for these comparisons in the section *Metrics* below.

We pick the model parameter values for our synthetic data using tighter priors than those presented in Table 1. The benefits of this approach are twofold. First, we avoid drawing simulations at the edges of the regions covered by the grids. Secondly, by choosing different priors, we select points that differ from those that enter the training data, although we use a Sobol sequence to sample the parameter space.

In the section *Results*, we infer the properties of our synthetic data using various techniques and compare the results with the ground truth. This comparison amounts to a self-consistent test allowing us to assess the performance of the different inference algorithms. However, due to the distance measure and likelihoods associated with the employed Bayesian inference techniques, i.e. ABC and MCMC, not all the synthetic data might be equally suitable for our purposes (cf. the sections *Rejection ABC* and *MCMC*). Especially when one or more of the output variables consistently take on a fixed value, the defined distance measure and likelihood functions run into trouble since we end up dividing by zero. One such scenario would be the case where the parameter values ensure that all tumour cells consistently die off before *t* = 100. Another example is the case where the disease is never passed on. To mitigate this problem, one might discard all synthetic data that produce delta functions as the marginals for one or more output variables of the ABMs—in the real world, these scenarios would not be studied, anyway. While these scenarios are certainly of mathematical interest, they are not of biological interest, at least for dealing with our synthetic data. Here, we apply a more conservative criterion, discarding all synthetic data, for which the width of the 68 per cent confidence interval is zero. With this selection criterion, we are left with 219 and 227 points in the parameter space for the cancer CA and epidemiological SIRDS models, respectively.

### Emulators

The purpose of an emulator is to mimic the output of the ABM at a low computational cost when given a set of ABM input parameters, often called features. Like the original ABM, the emulator is thus a function *f*(**Θ**) that maps from the model parameter space (**Θ**) to the output space of the ABM. The emulator can then be used as a surrogate model to sample the posterior probability distributions of the model parameters using likelihood-free inference (LFI) or other Bayesian inference techniques (cf. the sections *Rejection ABC* and *MCMC*).

Since the ABMs in this paper are driven by stochastic events, several simulations with the *same* input parameters will produce a distribution for each output variable of the ABM rather than a unique deterministic result. The emulator must capture this behaviour. Based on this notion, we follow three different approaches: We emulate the simulation output using a deep neural network (NN), a mixture density network (MDN), and Gaussian processes (GP).

We train and validate the emulators based on precomputed grids of models (cf. the section *Model grid*). When the ML methods rely on neural networks (NN and MDN), 60 per cent of the simulations are used for training, while the remaining 40 per cent are used to validate and test the methods before they encounter the synthetic data (cf. the sections *Synthetic data* and *Results*). For the Gaussian processes, 20 per cent are set aside for a testing phase, while the remaining 80 per cent of the simulations are used for training—when dealing with neural networks, the validation set is also needed for invoking early stopping during training. We also note that the different ML approaches do not see the *raw* synthetic data. Since we are working with outputs on different scales, the synthetic data are standardized before handing them over to the ML schemes.

#### Neural networks

As our first approach, we construct a broad, deep neural network (NN) with three hidden layers that contain 100 neurons each. To avoid overfitting, we include early stopping and dropout during the training phase [36]. The latter property implies that we randomly select 20 per cent of the neurons in each layer and temporarily mask these neurons during each pass through the NN [36]. To recover the stochastic nature of the ABMs, we maintain dropout during the inference phase and use the loss function proposed by Gal & Ghahramani [16, 37, 38]. These temporal changes in the NN’s architecture mean that the predictions of the NN become samples from a distribution rather than being deterministic. The resulting distribution encodes both epistemic and aleatoric uncertainties, i.e. the errors stemming from the limited knowledge of the NN and those stemming from the stochastic processes intrinsic to the ABMs. NNs that employ dropout have been shown to approximate Gaussian processes [16] and can be seen as an approximation of Bayesian neural networks [17]. Our implementation builds on TensorFlow [39].

#### Mixture density networks

As our second approach, we employ a mixture density network (MDN) that fits a mixture of multivariate normal distributions to the output of the ABMs [20, 21]. By sampling from the constructed mixture model during the inference process, it is possible to mimic the stochasticity of the underlying ABM.

The fit is accomplished by training a neural network to predict the means and full covariance matrices for each normal distribution. We use three components for each mixture model. For a five-dimensional space of output variables from the ABM, the neural network is thus optimized to predict 63 variables: 15 means, 15 diagonal and 30 unique off-diagonal elements of the covariance matrix, and 3 component weights. To ensure that the covariance matrices are positive semi-definite, we use exponential and linear activation functions for the diagonal and off-diagonal elements, respectively [40]. To guarantee that the component weights are positive and sum to unity, they are meanwhile passed through a softmax activation function. Regarding the loss function, we maximize the log-likelihood that the mixture model attributes to the training data from the grid discussed in the section *Model grid*. We employ early stopping and dropout like in the section *Neural networks*. Our implementation builds on TensorFlow [39].

#### Gaussian processes

Finally, as our third approach, we describe the output of the ABM as a Gaussian process (GP) [18, 41]. For a comprehensive overview of GPs, we refer the reader to work by Rasmussen et al. [19].

We use a linear combination of a radial basis function (RBF) kernel and a white noise kernel to specify the covariance of the prior function. We do so to account for the spread in the underlying output from the ABM [42]. Just as in the case of the MDN, the stochasticity of the ABM can be emulated during inference by sampling from the obtained GP.

Here, we set the mean squared error in the prediction to be the loss function, which amounts to the simplifying assumption that the output variables of the ABM are uncorrelated. We use the python implementation of Gaussian process regression from scikit-learn [43].

We note that the employed implementation produces a uni-modal distribution for a unique set of input parameter values. The same holds for the NN. In contrast, the MDN can produce multi-modal outputs when given a single input vector.

#### Rejection ABC

Having constructed an emulator, we can resort to standard sampling techniques to map the posterior probability distributions of the model parameters (**Θ**) of the ABM given a set of observations (***x***). In our case, these observations constitute the synthetic data created by the ABM as discussed in the section *Synthetic data*.

One option is to draw on the class of Approximate Bayesian Computation (ABC) methods [44–46]. The simplest among these algorithms is rejection ABC. Rejection ABC randomly draws samples from the prior probability distributions of the model parameters. The algorithm then compares the simulation output, i.e. the predictions of the emulator, with the observations using a suitable distance measure. Samples within a distance *δ* are kept, while the rest are discarded. In practice, *δ* is chosen in such a way as to keep a certain fraction of the samples. The distribution of these samples is then taken as a proxy for the true posterior.

In this paper, we use the implementation by Lintusaari et al. [47] (elfi). As regards the prior, we use uniform priors corresponding to the region covered by the grids discussed in the section *Model grid*. As regards *δ*, we keep the best 10^4^ of 10^7^ samples for each combination of parameters for our synthetic data.

The distance measure is the last thing we need to specify for the ABC. To motivate our choice, we need to consider the nature of the real-world applications that our ABMs strive to model. At best, a targeted laboratory study of glioblastoma or disease transmission may involve a handful of animals, such as mice or ferrets [48, 49]. Due to the limited number of specimens in the experiment, it can be notoriously complicated to assess the stochasticity that underlies the data. The same holds true when dealing with patient-specific data or data from the outbreak of an epidemic [50].

For instance, consider an experiment in which we implant human tumour cells in a single mouse to study tumour growth. Based on this time series alone, we cannot hope to rigorously constrain predictions for the outcome when repeating this experiment. Even given a handful of such experiments, we might be hard-pressed to go beyond simplifying assumptions, such as the notion that the observations will be normally distributed. Indeed, one might often not have enough information to go beyond measures, such as the median, the mean, the standard deviation, the standard deviation of the mean, the mean absolute error and/or the median absolute error. With this in mind, we settle for a distance measure (*d*) in the form

$$\begin{array}{c} d=\sum_{i}\frac{{\left[f{\left(\Theta \right)}_{i}-x_{\text{sim},i}\right]}^{2}}{\sigma_{\text{sim},i}^{2}}, \end{array}$$ (2)

where the sum runs over all five observed output variables of the ABM, *x*_sim,*i*_ is the median of the marginal distribution for the *i*th summary statistics in the synthetic data, and *σ*_sim,*i*_ denotes the standard deviation of the synthetic data. The distance is computed for each prediction made by the emulator denoted by *f*(**Θ**).

We note that *x*_sim,*i*_ and *σ*_sim,*i*_ do not fully capture the distribution of the synthetic data. In other words, by employing Eq (2), we assert the notion that real-world data do not reveal the same level of detail regarding the stochasticity of the studied events as our synthetic data do. This being said, we also note that the marginal distributions obtained from the synthetic data are mostly uni-modal and relatively symmetric distributions. The simplifying assumptions that enter Eq (2) thus still give a reasonable depiction of the underlying synthetic data.

#### MCMC

Likelihood-free inference (LFI) algorithms, such as rejection ABC, lend themselves to our analysis because the likelihood that describes the system is intractable. By introducing a distance measure based on suitable summary statistics, rejection ABC thus altogether avoids the necessity of consulting the likelihood when inferring the posterior distribution.

Alternatively, one might introduce a surrogate likelihood. This approach is, indeed, commonly used across different fields of research [51, 52].

Following similar arguments to those that lead to the distance measure in Eq (2), we arrive at a surrogate likelihood, i.e. *p*(***x***|**Θ**), in the form

$$\begin{array}{c} \text{log}\;p\left(x|\Theta \right)=\sum_{i}\frac{{\left[f{\left(\Theta \right)}_{i}-x_{\text{sim},i}\right]}^{2}}{\sigma_{\text{sim},i}^{2}}, \end{array}$$ (3)

where *x*_sim,*i*_ and *σ*_sim,*i*_ again denote the median and standard deviation of the marginal of the *i*th output variable in the synthetic data, respectively.

However, the use of Eq (3) leads to convergence problems due to the stochastic nature of the ABMs. Alternatively, one might directly compare the predicted median (*M*) with that of the observations. To account for the stochastic behaviour of the ABMs, we include the standard deviation (*σ*_pred_) of the prediction in the denominator:

$$\begin{array}{c} \text{log}\;p\left(x|\Theta \right)=\sum_{i}\frac{{\left[M\left(f{\left(\Theta \right)}_{i}\right)-x_{\text{sim},i}\right]}^{2}}{\sigma_{\text{sim},i}^{2}+\sigma_{\text{pred},i}^{2}}. \end{array}$$ (4)

We refer to the surrogate likelihoods in Eqs (3) and (4) as case a and b, respectively.

Having defined a surrogate likelihood, we can employ standard sampling techniques, such as Markov chain Monte Carlo (MCMC) [6, 53]. The advantage of this approach over rejection ABC is that we can sample more efficiently from the prior. However, as discussed in the section *Results* and mentioned above, the stochasticity of the emulator, partly reflecting the stochasticity of the ABM, might compromise convergence.

This paper uses the ensemble sampler published by Foreman-Mackey et al. [54] based on the procedure suggested by Goodman & Weare [55]. We thus evolve an ensemble of *N* walkers in parallel, where *N* is twice the number of dimensions of the mapped input parameter space of the ABM. For each combination of parameters in our synthetic data, we exclude a burn-in phase containing 5000 samples per walker. We then collect 20,000 additional samples per walker. We note that this approach does not imply that we obtain 20,000 truly independent draws from the distribution. The number of samples necessary for the walker to become oblivious to its initial conditions is called the integrated autocorrelation length. To assess the robustness of the MCMC, we thus estimate this quantity. For further details, we refer the interested reader to the paper by Foreman-Mackey et al. [54].

### Direct inference

As discussed above, emulators mimic the ABMs by producing samples from the same output variable space (***x***) when given a set of parameter values (**Θ**). To infer the parameter values that correspond to a set of observations using emulators, we sample the parameter space using Bayesian sampling schemes and compare the output of the emulators to the observations (cf. the flowchart in Fig 1). When using emulators in inference, we thus go from the input space of the ABMs to their output space and back. Alternatively, we can construct an ML algorithm that directly predicts the parameter values (**Θ**) given points from the output variable space (***x***) of the ABMs. In the following, we will refer to such an algorithm as a direct inference machine.

Since direct inference machines take us directly from the observations to the parameter values, they do not rely on Bayesian sampling schemes, such as ABC or MCMC [12, 13]. Once direct inference machines are trained, they are thus much faster to employ than emulators. In the results section, we quantify this statement. However, this advantage comes at the cost of lower flexibility and reduced transparency.

While it is thus straightforward to determine the performance of an emulator by assessing its ability to mimic the output of the simulations, direct inference machines do not lend themselves to this type of assessment since they produce predictions of the underlying parameter values without any such intermediate steps. We must fully rely on performance measures that assess the final predictions for **Θ** (cf. the section *Metrics*). In this sense, direct inference machines are less transparent than emulators.

Having established an emulator, it is also straightforward to tweak the priors or alter the likelihood function or distance measure during the inference stage, i.e. using the ABC or MCMC. It is only necessary to re-train the emulator if we extend or change the parameter space we explore. On the other hand, each such change would require the ML algorithm to be re-trained when using direct inference machines. In this sense, direct inference machines are less flexible than emulators. Of course, this property only becomes an obstacle for direct inference machines if the training phase is a computational bottleneck, which is not the case for our examples.

Both emulators and direct inference machines thus have their advantages and shortcomings. Rather than resorting to a one-size-fits-all approach, one must choose between different algorithms based on the inference problem one faces. Indeed, both emulators and direct inference machines are very popular in different research fields [13, 21, 34].

In this paper, we compare the results obtained using the emulators with those obtained using two direct inference machines. We employ a broad, deep neural network as our first direct inference technique. Apart from the fact that the input and output spaces are swapped, the architecture of this neural network matches that described in the section *Neural networks*. As our second approach, we train a Gaussian process based on the settings summarized in the section *Gaussian processes*. Like the emulators, the direct inference machines account for the stochastic processes intrinsic to the system through our choice of machine learning methods and through the training of these algorithms.

Before commencing, we still need to address one question: How do we establish credibility intervals for the direct inference machine? Consider the case where we repeatedly generate samples using one of our direct inference machines based on the *same* set of singular values for each of the input variables. The chosen direct inference machine would then yield a distribution that reflects the intrinsic stochastic properties of the training data, i.e. the ABMs, and the limitations of the information captured by the training data. However, we also need to include the uncertainty of the observations. Our synthetic data are thus distributions rather than singular values, and we should not run the direct inference machine repeatedly using the *same* set of input variables. Rather, to include the observational errors, we would need to generate samples from a distribution of input parameters that corresponds to the distribution of the observations [34]. As discussed in the section *Rejection ABC*, we assume that independent Gaussian distributions approximate the marginal distributions of the observed quantities. Following the outline by Bellinger et al. [34], we thus sample 10,000 combinations of input parameters for the direct inference machines using a multivariate Gaussian distribution with a diagonal covariance matrix matching the observational constraints. The means of this multivariate distribution are set to the medians of the observations, while the variance reflects the standard deviations of the marginals.

### Metrics

Due to the stochastic nature of the ABMs, we arrive at distributions for their output variables when repeating runs with the *same* input parameters. We call on four measures to quantify how well our emulators recover these distributions. For each measure, we compare properties of the marginal distributions predicted by the emulators to those obtained from the simulations, i.e. from the ground truth of the synthetic data:

- As our first measure, we compute the error in the predicted means of the marginal distributions, i.e. *μ*_pred_ − *μ*_sim_. Here, the subscripts ‘pred’ and ‘sim’ refer to the predictions by the emulators and the results obtained from simulations, respectively. Any deviations by the mean of this measure from zero would reveal a bias. Since we cover a broad region of the parameter space for each ABM, we cite this measure in units of the standard deviation of the marginal obtained from the simulations (*σ*_sim_).
- As our second measure, we include the relative absolute error in the predicted median, i.e. |*M*_pred_ − *M*_sim_|/*M*_sim_. Like the first measure, this measure quantifies the accuracy of the emulator.
- Thirdly, we compute the ratio between the predicted standard deviation of the marginal distribution and the standard deviation obtained from simulations (*σ*_pred_/*σ*_sim_). This measure should be close to one. Otherwise, the emulator over- or underestimates the uncertainties of the predictions.
- Finally, we compute the Wasserstein distance that quantifies the discrepancy between probability distributions [56]. The lower this measure is, the more similar the predicted distribution by the emulator is to the ground truth.

We note that the metrics chosen for the output of the emulators reflect our assumptions about the nature of the data that we would obtain in a real-world scenario. As elaborated upon in the section *Rejection ABC*, we thus assume the observations to be approximated by a multivariate Gaussian distribution with a diagonal covariance matrix. Although we deem the listed measures to be the most suitable, it is still worth stressing that generalisations of the above measures and alternative measures exist [13].

Meanwhile, the assumption that the observations are normally distributed does not affect the metrics with which we assess the inference results, i.e. the obtained posterior distribution for the model parameters. The marginal posterior distributions might very well be skewed or multi-modal, and the metrics should be able to handle this. We turn to these metrics below.

As regards the ABC runs, the MCMC runs, and the direct inference machines, we can compare the obtained distributions to the original input parameter values (**Θ**_o_) of the synthetic data. To assess the performance of the algorithms, we thus compute both the residuals and relative difference between the mean of the predicted marginal distributions and the corresponding elements (*θ*_o_) of **Θ**_o_. In addition, we compute

- the negative logarithm of the probability density, *q*(*θ*_*o*_), that is attributed to the true value by the algorithm. The average, $-E[\text{log}\;q\left(\theta_{o}|x_{o}\right)]$, of this measure across input and output variables (**Θ**_o_, ***x***_o_) is widely used in the literature [13, 57]. The lower the value of −log *q*(*θ*_*o*_) is, the better the algorithm is at recovering the ground truth. Note that this metric does not build on any assumptions regarding the shape of the marginal posterior distributions.

These measures quantify the accuracy and precision of the considered inference techniques. By comparing the measures across the different algorithms, we can put the performance of the individual algorithms into perspective.

As an additional comparison, one might propose to infer the model parameters using the actual simulations in tandem with the ABC or MCMC algorithms. We could then use the obtained distributions as benchmarks. However, since we are dealing with complex ABMs, any analysis involving the actual simulations becomes insurmountable in terms of computational resources. After all, this stumbling block was the reason for turning to emulators and direct inference machines in the first place.

## Results

As discussed in detail in the method section above, we use two main approaches to simulation-based inference: emulation-based approaches and direct inference machines. We employ three different emulators: a deep neural network (NN), a mixture density network (MDN), and Gaussian processes (GP). These methods were chosen because they can mimic the stochastic nature of the ABMs. The predictions of the emulators are directly compared to the synthetic data, i.e. the synthetic data (cf. the section *Synthetic data*). We then infer the underlying model parameters (**Θ**) using rejection ABC and Markov Chain Monte Carlo (MCMC). Finally, we compare these ML predictions with the ground truth (**Θ**_o_). For the ABC, we employ a single distance measure that compares individual predictions to the synthetic data, while we take two distinct approaches for the MCMC: The likelihood either relies on individual predictions (case a) or global properties of the predicted distributions (case b).

Regarding the direct inference machines, we again employ a NN and GP and compare the predictions to the ground truth. For each method, we vary the size of the training set to evaluate its impact on the results. Further details are given in S1 Appendix. For a concise overview of the different steps, we refer the reader to the flowchart in Fig 1.

### Comparing emulators

When comparing the predictions of the emulators to our synthetic data, we consider the absolute error in the mean, the relative error in the median, the ratio between the predicted and true width of the distribution in terms of the standard deviation, and the Wasserstein distance between predicted and true distribution (see methods section for details).

**Fig 2:**
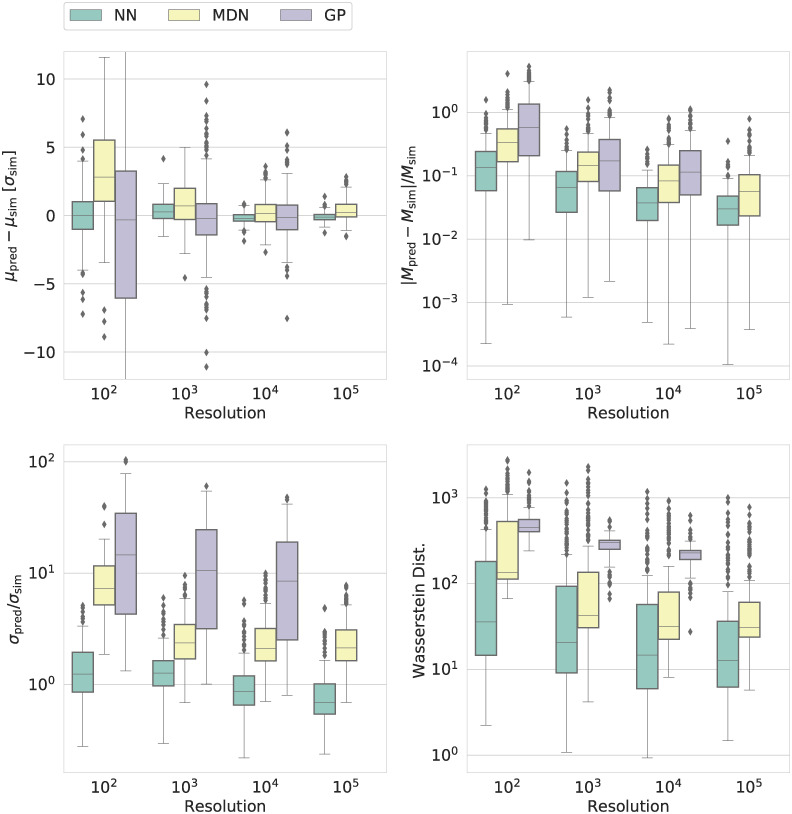
Comparison of the performance of the emulators as a function of the size of the training set (resolution) for the agent-based cellular automata (CA) model of brain tumours. The plot shows the results for three emulators: A neural network (NN), a mixture density network (MDN), and a Gaussian process (GP). The subscripts ‘pred’ and ‘sim’ refer to the predictions by the emulators and the ground truth obtained from simulations, i.e. the synthetic data, respectively. The four panels contain our four performance metrics for emulators: the error in the predicted mean (*μ*_pred_), the relative absolute error in the predicted median (*M*_pred_), the ratio between the predicted width of the marginals in terms of the standard deviation (*σ*_pred_) and true width, and the Wasserstein distance (cf. the section *Metrics*). The figure summarizes these quantities for one of the output variables of the CA model, the number of glioblastoma stem-like cells (GSC). For the other outputs, we refer the reader to S7 Fig.

**Fig 3:**
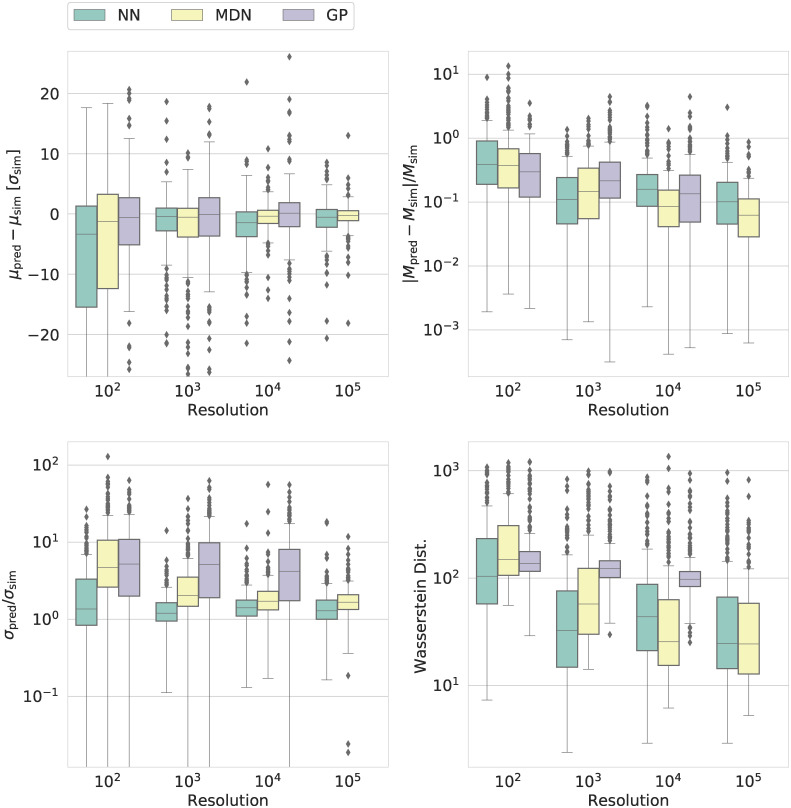
Counterpart to Fig 2 for our epidemiological SIRDS model. The plot shows different metrics of the performance of emulators for one of the output variables of the SIRDS model, the total duration of the epidemic. For the other outputs, we refer the reader to S8 Fig.

Figs 2 and 3 (as well as S7 and S8 Figs) summarize the comparisons between the emulation-based predictions and the distributions obtained from simulations for the ABMs describing tumour growth and disease spread, respectively. Our ABM describing brain cancer is a cellular automata (CA) model (cf. methods section). Our epidemiological model is a SIRDS model (cf. the methods section). In the following, we refer to the ABMs using this terminology.

As regards the cancer CA model for tumour growth, the NN outperforms the other ML techniques across nearly all measures, sizes of the training set, and variables. The NN closely recovers the correct mean, median, and width of the marginal distributions obtained from simulations and generally yields the lowest Wasserstein distance. In contrast, the MDN is generally biased, overestimating the true mean, while the GP results in much too broad distributions.

For our epidemiological SIRDS model, on the other hand, the MDN outperforms the NN for sufficiently large training sets. The GP still yields the worst performance across all measures.

As can be seen from Figs 2 and 3 (as well as S7 and S8 Figs), the accuracy and precision of the different ML approaches generally improve with increasing size of the training set. However, the GP cannot cope with the kernel matrix for the largest training set. This is not to say that this is a general flaw of Gaussian processes [58] but rather goes to show that the suitability of any ML implementation or approach is problem-dependent.

### Comparing inference techniques

When comparing the inference results to the ground truth, we include four metrics: We compare the mean of the distribution to the true parameters and compute both the relative and absolute error. Moreover, we consider the standard deviation of the posterior and the probability density (*q*) attributed to the true value (see further details on metrics used in the method section).

As mentioned above, the NN and GP result in the best and worst performance among the explored emulators for the cancer CA model, respectively. To see what impact this performance gap has on the inference, we sampled the parameter space using both emulators in tandem with both ABC and MCMC. Due to the associated computational cost, we do not repeat this analysis for different sizes of the training set. Instead, we settle for a resolution of 10^4^ simulations in the training set, as the NN performs well at this resolution. Any additional gain in performance at 10^5^ simulations does not outweigh the substantially increased computational expense. For our epidemiological SIRDS model, we limit ourselves to the NN as regards the emulation-based inference.

**Fig 4:**
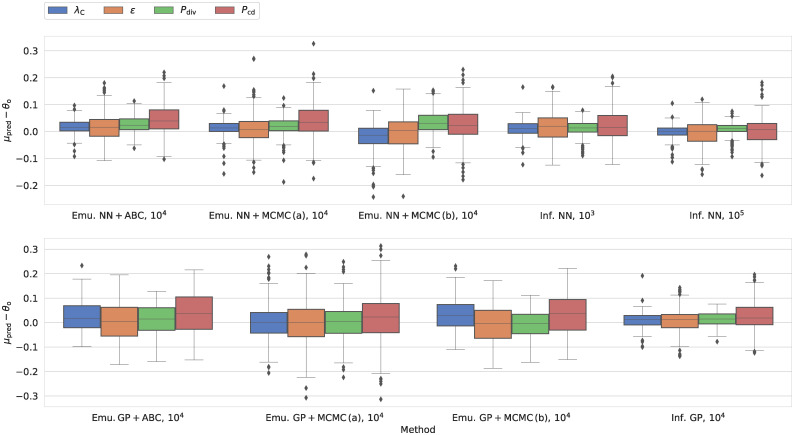
Performance across different inference schemes for our cancer CA model in terms of the residuals between the mean of the marginal distribution and the true parameter values (*θ*_o_). For other metrics, we refer the reader to S9 Fig. The plot includes the results from the emulation-based approaches (emu.) and our direct inference machines (inf.). We include two machine learning approaches: a neural network (NN) and Gaussian processes (GP). In connection with the emulators, we distinguish between results obtained using rejection ABC and MCMC. For each approach, the label specifies the size of the training set: For the emulators, we consistently used 10^4^ simulations.

For the cancer CA model, the higher accuracy and precision of the NN emulator are reflected in the performance metrics for the inference results. We illustrate this result in Fig 4 (as well as S9 Fig). Whether we use the ABC or MCMC algorithm for inference, the NN leads to a narrower distribution, whose mean more closely recovers the ground truth than the GP.

To put the results in Fig 4 (and S9 Fig) into perspective, consider the case where we use the mean of the posterior distribution as our parameter estimate. This approach would result in unbiased residuals for the mean with 25th and 75th percentiles at -0.1 and 0.1, respectively. All methods thus perform better than a random assignment of parameter estimates.

Irrespective of the ML approach, the MCMC (cases a and b) and ABC lead to similar values of the four measures, including −log *q*(*θ*_*o*_). However, due to the stochastic nature of the emulator, we note that the median autocorrelation lengths across all four input parameters are 383–412 and 1889–1953 samples for NN and GP MCMC runs, respectively, when individual predictions by the emulator are used in the likelihood (case a). We summarize these numbers in Table 2. The GP emulator thus results in a lower number of effective samples. Indeed, for the GP, the resulting contour plots are very noisy, often consisting of disconnected peaks. This notion calls the robustness of the MCMC results based on the GP into question. We attribute this to the fact that the GP emulator overestimates the standard deviation for the output parameters of the ABM. This increased stochasticity hinders convergence.

**Table 2:** Median and mean autocorrelation length for different MCMC setups. For each of the two ABMs (our cancer CA and epidemiological SIRDS model), we use two ML emulation algorithms (NN and GP). We distinguish between two different likelihoods (case a and b, cf. the methods section). The first column specifies the different setups by first naming the ABM, followed by the emulator and the likelihood.

MCMC	Mean	Median
CA-NN (a)	565–599	383–412
CA-NN (b)	377–402	163–174
CA-GP (a)	1835–1875	1889–1953
CA-GP (b)	423–456	311–346
SIRDS-NN (a)	2084–2120	2118–2179
SIRDS-NN (b)	325–393	194–266

**Fig 5:**
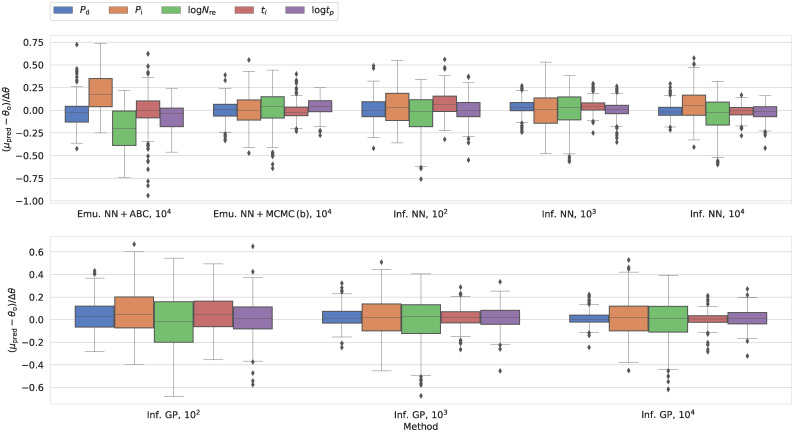
Counterpart to Fig 4 for our epidemiological SIRDS model. Here, we show the performance across different inference schemes for our SIRDS model in terms of the residuals between the mean of the marginal distribution and the true parameter values (*θ*_o_) in units of the width of the observed parameter range (Δ*θ*). In contrast to Fig 4, we scale the residuals based Δ*θ* because the different parameters cover very different ranges. For other metrics, we refer the reader to S10 Fig.

As regards the epidemiological SIRDS ABM, the case a of the MCMC algorithm suffers from the same issue, even when dealing with emulators that closely recover the marginal distributions obtained from simulations. For the NN MCMC runs, the mean and median estimated autocorrelation lengths across the different parameters lie between 2084 and 2179, and the associated contour plots are extremely noisy when individual predictions by the emulator are used in the likelihood (case a). Due to this behaviour, we have omitted case a of the MCMC results in Fig 5 (and S10 Fig) that summarizes the performance of the different inference techniques for the epidemiological SIRDS ABM.

Rejection ABC, on the other hand, does not suffer from the same problem for either of the two ABMs. Even though rejection ABC leads to high values of −log *q*(*θ*_o_) in some cases, this technique can reliably reconstruct the mean and median. Moreover, in contrast to the MCMC (case a) that leads to spurious contour plots, rejection ABC does not yield equally noisy posteriors even though the distance measure draws on individual predictions by the emulator. To improve the contour plots of the MCMC, we have to resort to using global parameters of the predicted distributions rather than individual samples in the likelihood (case b). When doing so, the median autocorrelation length lies in the intervals 163–174 and 194–266 for the cancer CA and epidemiological SIRDS models, respectively (cf. Table 2).

Figs 4 and 5 (as well as S9 and S10 Figs) also include the predictions by the NN and GP direct inference machines based on different sizes of the training set. Two features stand out: First, the direct inference machines perform as well or better than the emulation-based approaches, even with a low number of simulations in the training set. Indeed, some measures do not visibly improve even when the model is confronted with significantly more training data. Secondly, the dissonance in −log *q*(*θ*_*o*_) between the NN and GP is more prominent for the epidemiological SIRDS ABM than in the case of the cancer CA model.

As regards both ABMs, it bears mentioning that the values obtained for the four metrics are partly correlated. However, the correlations are method- and measure-dependent. They might thus be erased when comparing samples across different methods and measures, e.g. reflecting differences in the performance and the normalization factors.

All inference techniques struggle with the same model parameters (e.g. log *N*_re_ for the SIRDS model), while they are all reasonably good at establishing certain other model parameters (e.g. *P*_d_ for the SIRDS model). This behaviour is hardly surprising. It merely reflects the non-identifiability of certain parameters given the available information through the summary statistics. While one might overcome this issue by choosing different summary statistics, we note that this might not be straightforward in real-world applications—e.g. due to limitations imposed by the data.

### Performance analysis

**Fig 6:**
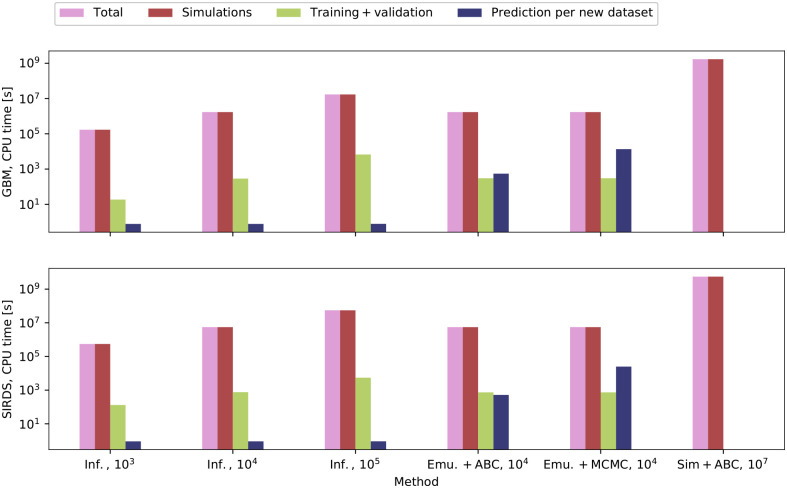
Required CPU time across different methods for our cancer CA model (upper panel) and the epidemiological SIRDS model (lower panel). The light magenta bars show the total time required for each inference technique. The remaining three bars specify the time required for the simulations involved (red), the time required for training and validation (yellow), and the time that is needed to infer the parameters for a single set of observations (blue). The number of required simulations is specified in the labels. We include the NN direct inference machine based on three different sizes of the training set. Moreover, we include the NN emulator in tandem with the rejection ABC and MCMC algorithms. The ABC accepts the best 10^4^ among 10^7^ randomly drawn samples. The ensemble MCMC uses 8 walkers, drawing 20,000 samples for each walker. For the MCMC, we average over the CPU times for cases a and b. For comparison, we include the estimated time required for running the ABC with 10^7^ simulations. Note that the ordinate is logarithmic. Note also that the time needed to run any given simulation depends on the number of agents and the model parameters.

To quantify the motivation for using the different inference techniques, we summarise the CPU time requirements for each method in Fig 6. Since computation time is greatly model-dependent, our intention is thus merely to underline a few key features.

As shown in the figure, the computation of the simulations consumes the most resources: The total time in every case is almost equal to the simulation time. Training the direct inference machines or emulators takes orders of magnitude less time, and new simulations are only required during the training phase.

To further substantiate the reduction in computational cost associated with our approach, consider an MCMC run based on the likelihood in Eq (3). Since we use the ensemble sampler by Foreman-Mackey et al. [54], each run involves twice as many walkers as the dimensions of the output parameter space, i.e. 8–10 walkers. Each walker leads to a Markov chain containing 25,000 samples, including the burn-in. For eight walkers, each MCMC run thus requires 200,000 individual samples. If we were to use actual simulations rather than the emulators, we would thus need 200,000 simulations for each MCMC run. To fit the 219 synthetic synthetic brain cancer data sets discussed in this paper, we would need 43.8 million simulations for the MCMC runs based on Eq (3) alone. To fit the 227 synthetic epidemiological data sets, we need 45.4 million simulations. Even more samples are required to perform the analysis based on Eq (4) or the ABC algorithm. In contrast, we demonstrate that only 10,000 simulations are sufficient to train *all* our emulators and direct inference machines for both the CA and SIRDS models. Our machine learning methods can thus reduce the number of simulations needed by several orders of magnitude.

For our models, the training of the emulators and direct inference machines take the same time, which does not always have to be the case. However, the direct inference machines are more effective in obtaining estimates for the underlying model parameters. Moreover, since the simulations take the most time, investing in training more complex ML emulators or direct inference machines will not be more computationally expensive and may pay off in the accuracy of the results.

## Discussion

This paper investigates how to efficiently perform inference when relying on computationally expensive stochastic agent-based models (ABMs). For this purpose, we compute synthetic data and infer the underlying model parameters in self-consistent tests using various techniques. These methods fall into two main categories: emulators and direct inference machines. We find that the performance of the emulators and direct inference machines are not consistent across the two ABMs but rather depend on the real-world application in question and the size of the training set. They thus depend on the available computational resources. This leads us to our first guideline:

- There is no one-size-fits-all solution for simulation-based inference among the presented ML methods. The performances of the tested algorithms are problem-dependent. Since there are *a priori* no clear-cut preferences, we recommend **confronting data with several techniques and selecting the best method based on suitable metrics** rather than relying on a pre-selected ML algorithm or other surrogate models.

We do not present an absolute ranking of the methods per se but rather raise caution about solely relying on a specific method when facing a new research problem. We do, however, note that the GP performed worse than the NN and MDN methods. So, one may need to be careful when relying on Gaussian processes for emulation [59–61]. These results are worth highlighting, as Gaussian processes are often the go-to approach. This being said, other implementations of Gaussian processes might be able to address the shortcomings of the implementation used here [62, 63].

The notion that different methods may work better for different problems is consistent with the findings by Lueckmann et al. [13], who study a range of benchmark problems to provide guidelines for sequential inference techniques. They do likewise not find a clear-cut preference among their sequential algorithms. In a nutshell, sequential techniques explore the parameter space iteratively. Rather than relying on a static grid for training, they hand-pick those points in the parameter space that closely match the specific observation whose properties we aim to infer. Sequential techniques can, as a result of this, greatly reduce the number of required simulations by orders of magnitude. Implementations for both emulation and direct inference exist [64, 65]. The catch is that new simulations are required for every new set of observations. With 250 test cases for each ABM in our analysis, we can thus not reap the benefits of these algorithms as a substitute for our inference machines. However, sequential techniques have proven very powerful in fields such as cosmology [40]. After all, there is only *one* universe.

When comparing our results to those by Lueckmann et al., it is also worth noting that we use an alternative application of neural networks for emulation and direct inference in this paper: Rather than estimating the likelihood or posterior densities, we use a neural network with dropout that provides an approximate Bayesian formulation and mimics a Gaussian process [16, 17, 37, 38]. Like the methods discussed by Lueckmann et al., we find this alternative approach very effective in producing good emulators and direct inference machines with a relatively small number of simulations sampled from the parameter space in our examples.

Yet another distinct aspect of our study is that we keep the limitations of the ABM applications in mind: When dealing with real-world data, one must often resort to simplifying assumptions regarding the true distribution of the data due to the nature of the experiments. Not only does this dictate the type of data we can gather, but it also imposes constraints on the summary statistics and the metrics we can draw on (cf. the methods section). Our paper outlines how to select different methods and performance measures based on the research problem using ABMs as a case study.

We do not claim that our list of emulators and direct inference machines is exhaustive. Other viable approaches exist with scope for future developments. For example, recent work on generating differential equations from stochastic ABMs [66] might be extended to stochastic settings to generate new emulators. However, it is beyond the scope of the present paper to explore all possible paths. Rather, we intend to present an illustrative selection. We also note that not all ML methods can readily address the stochastic nature of the data. At least not in their standard form. Acquiring error bars might require elaborate extensions (cf. the paper by Meinshausen et al. [67] for a discussion on regression random forests [68]). Moreover, as mentioned in the introduction, one might draw on other surrogate models, such as mathematical parameterizations or mechanistic models, from outside the realm of machine learning algorithms.

For our ABMs, we find that the direct inference machines provide accurate and precise results even with minimal training. Since computation time is one of the most prominent obstacles when dealing with ABMs for real-world applications, this feature favours direct inference algorithms. Thus,

- we generally recommend to **look for techniques that minimize the number of simulations required** such as direct inference machines.

This is not to say that direct inference machines will be the optimal choice for all ABMs. The present success of the direct inference machines might reflect aspects such as the complexity of the true posterior distributions or the adequacy of the distance measure and likelihood used by the statistical inference technique, i.e. rejection ABC and MCMC. However, we note that the presented direct inference machines (and the emulation-based ABC or MCMC) can produce multi-modal posteriors.

As regards the statistical inference techniques, we find that the MCMC does not always yield robust results. We attribute this to the stochastic nature of the ABMs. While the rejection ABC consistently yields worse results than the direct inference machines in terms of the employed metrics, it does not suffer from this shortcoming.

- Due to the stochastic nature of the agent-based models, **some statistical inference techniques do not yield robust constraints for emulation-based approaches**.

The statistical algorithms explored in this paper, i.e. rejection ABC and MCMC, are not exhaustive. Other viable alternatives exist, such as sequential Monte Carlo (SMC) [69]. The aforementioned sequential techniques discussed by Lueckmann et al. offer another alternative when combined with the emulators. However, we also note that the stochastic nature and intractable likelihood of the explored systems render some approaches impractical. For instance, while Hamiltonian Monte Carlo (HMC) [70] is generally a demonstrably powerful technique [71], it is not suited for our purposes as we cannot reliably compute derivatives in the explored parameter space.

In connection to this, it is also worth pointing out that grid-based approaches, such as the ones used here, do not scale very well to high-dimensional parameter spaces unless the relevant summary statistics show limited variation across the space. Otherwise, a vast number of simulations is required. However, grid-based approaches are commonly employed in the literature because they are transparent and less convoluted than other methods. When dealing with higher-dimensional spaces, we also note that one might consider introducing effective means of dimension reduction [72], i.e. reducing the number of relevant parameters, or informing the ML methods about rules that govern the system [73].

By addressing and comparing multiple widely-used algorithms, we hope that the present paper assists researchers in selecting appropriate inference techniques for real-world applications. While we have focused on ABMs as one class of real-world models, we believe our results to be relevant to other stochastic complex systems. State-of-the-art numerical models are often computationally heavy, and we hope our guidelines and recommendations help modellers overcome this stumbling block. We have made our code available on https://github.com/ASoelvsten/ABM to facilitate this goal.

## Supporting information

S1 Appendix Supplementary methodology. The appendix includes a detailed discussion of our brain tumour CA model and our epidemic SIRDS model as well as an application of our ML methods to the Schlögl model.
(PDF)

S1 Fig Snapshots of tumours created using the brain cancer CA model. The **left panel** shows a tumour with *P*_div_ = 0.25, *P*_cd_ = 0.05, λ_C_ = 0.02 units of nutrients consumed per cell per time step, and *ϵ* = 0.2 at *t* = 280. Single GSC, GPP, and GDS are shown using purple, red and yellow markers, respectively. Dead tumour cells or clusters containing only dead cells are marked with black dots. Clusters with quiescent cells are blue. At this point, the tumour is spherical and its growth is well-described by a Gompertz curve. The **right panel** shows a tumour that differs only in that λ_C_ = 0.1 and in that the snapshot is taken at *t* = 1170. Due to starvation, higher mortality causes a more irregular growth pattern. In both panels, the cells live on an adaptive irregular lattice with up to 1,000,000 cell sites—we note that the simulations used throughout the paper are run in a smaller box (cf. S2 Fig). By comparing the left and right panels, we note that our emulators and direct inference machines are thus faced with tumours that show very different topology.
(EPS)

S2 Fig Example summary of the output of the brain cancer CA model for a single model run. The **left panel** summarises the tumour growths in terms of the size of the different cell subpopulations. For this run, *P*_div_ = 0.25, *P*_cd_ = 0.05, λ_C_ = 0.1 units of nutrients consumed per cell per time step, and *ϵ* = 0.2. We include the number of GSC, GPP, GDS, and dead tumour cells, as well as the total number of alive tumour cells and the number of alive tumour cells in the proliferating rim. The **right panel** shows a snapshot of the tumour at *t* = 100. The reaction-diffusion equation that describes the nutrient flow is solved on a Cartesian grid. This grid is highlighted in cyan outside the tumour. The cells live on an adaptive irregular grid that can, at most, harbour 40,000 cells. This adaptive grid is highlighted in cyan inside the tumour. In the proliferating rim, the irregular grid has a high resolution. In the bulk tumour, several cells share one lattice site. Single GSC (purple), GPP (red), and GDS (yellow) are shown using markers whose colours match those in the left panel. Dead tumour cells or clusters containing only dead cells are marked with black dots. Clusters with quiescent cells are blue. The presence of a nutrient source in the lower-left corner and the imposed cell death upon starvation introduce an asymmetric growth pattern.
(EPS)

S3 Fig Example summary of the output of the epidemiological SIRDS ABM for a single model run. The **left panel** summarizes the time evolution of the different subpopulations (*S*, *I*, *R*, and *D*) with 10,000 individuals randomly distributed over 12,100 lattice sites. In this scenario, *P*_d_ = 0.1, *P*_i_ = 0.2, (*N*_re_) = 5.1 random encounters per infected individual per day, *t*_i_ = 7.1 days, and *t*_p_ = 5000 days. For illustrative purposes, the **right panel** shows a snapshot at *t* = 6.0 days for a simulation with the same parameter values but only 1000 agents randomly distributed over 1225 lattice sites. The colour coding matches that in the left panel: cyan dots mark susceptible individuals, red dots mark infected individuals, recovered individuals are green, and black dots mark dead agents.
(EPS)

S4 Fig Comparison of the performance of the emulators across the parameter space for variables for the Schlögl reaction network (counterpart to S7 and S8 Figs). The plot shows the results for two emulators: a neural network (NN) and a Gaussian process (GP). The subscripts ‘pred’ and ‘sim’ refer to the predictions by the emulators and to the ground truth obtained from simulations, i.e. the synthetic data, respectively. Note that the comparison is conducted on a logarithmic scale, i.e. we deal with log_10_(*X*), log_10_(*A*), log_10_(*B*), and log_10_(*T*). From left to right, the panels contain the absolute error in the predicted median, the ratio between the predicted width of the marginals in terms of the standard deviation and true width, and the Wasserstein distance. Here, we only include the 141 test cases, for which *σ*_sim_ ≠ 0 for all species (the exclusion of the remaining 79 models is mostly due to *B*). Including all models does not change the picture as regards the median or the Wasserstein distance. However, the GP performs even worse for *σ*_pred_/*σ*_sim_ in the excluded cases.
(EPS)

S5 Fig Performance for two direct inference machines (inf.) for the Schlögl model. The first row contains the residuals between the mean of the marginal distributions and the true parameter values (**Θ**_o_) for each method. The second row shows the corresponding relative error in the prediction. The third row summarises the standard deviation of the marginals, while the fourth row shows the negative logarithm of the probability density (*q*) attributed to the true parameter value. Note that we compare *k*_1_-*k*_4_ with the ground truth on a logarithmic scale. We use a neural network (NN) and Gaussian processes (GP). For each approach, the label specifies the size of the training set.
(EPS)

S6 Fig Contour plot showing the variation in *X* and *T* across the parameter space spanned by *k*_1_, *k*_2_, *k*_3_ and *k*_4_ based on the training data. While both *X* and *T* exhibit clear contours in the planes spanned by *k*_1_ and *k*_2_, a noisier picture emerges in the planes panned by *k*_3_ and *k*_4_.
(EPS)

S7 Fig Comparison of the performance of the emulators as a function of the size of the training set (resolution) for the agent-based cellular automata (CA) model of brain tumours. The plot shows the results for three emulators: A neural network (NN), a mixture density network (MDN), and a Gaussian process (GP). The subscripts ‘pred’ and ‘sim’ refer to the predictions by the emulators and the ground truth obtained from simulations, i.e. the synthetic data, respectively. From left to right, the columns contain the error in the predicted mean (*μ*_pred_), the relative absolute error in the predicted median (*M*_pred_), the ratio between the predicted width of the marginals in terms of the standard deviation (*σ*_pred_) and true width, and the Wasserstein distance (cf. the Section *Metrics*). Rows A-E contain the number of glioblastoma stem-like cells (GSC), the number of cells in the propagating progeny (GPP), the number of cells in the differentiating subpopulation (GDS), the number of dead tumour cells, and the number of cells in the proliferating rim, respectively.
(EPS)

S8 Fig Counterpart to S7 Fig for our epidemiological SIRDS model. Rows A-E contain the total duration of the epidemic, the total death toll, the highest number of infected individuals during the outbreak, the time at which the peak in infections occurs, and the highest number of recovered agents during the outbreak, respectively.
(EPS)

S9 Fig Performance across different inference schemes for our cancer CA model. The first row contains the residuals between the mean of the marginal distributions and the true parameter values (**Θ**_o_) for each method. The second row shows the corresponding relative error in the prediction. The third row summarises the standard deviation of the marginals, while the fourth row shows the negative logarithm of the probability density (*q*) attributed to the true parameter value. The plot includes the results from the emulation-based approaches (emu.) and our direct inference machines (inf.). We include two machine learning approaches: A neural network (NN) and Gaussian processes (GP). In connection with the emulators, we distinguish between results obtained using rejection ABC and MCMC. For each approach, the label specifies the size of the training set: For the emulators, we consistently used 10^4^ simulations.
(EPS)

S10 Fig Counterpart to S9 Fig for our epidemiological SIRDS model. (EPS)
