# Supplementary material for: Efficient Bayesian inference for stochastic agent-based models
Source: PLoS Comput Biol. 2022 Oct 5;18(10):e1009508. doi: 10.1371/journal.pcbi.1009508 (PMC9576090; doi:10.1371/journal.pcbi.1009508)
Supplement: S1 Appendix — The appendix includes a detailed discussion of our brain tumour CA model and our epidemic SIRDS model as well as an application of our ML methods to the Schlögl model. (PDF) [file pcbi.1009508.s001.pdf]

S1 Supplementary Methodology

The figures discussed in this appendix to support the text are individually available as S1-S6 Figs.

Brain tumour, CA model

A myriad of complementary methods offer ways to construct spatio-temporal agent-based models for cancer. We kindly refer the interested reader to the review by Liedekerke et al. [1] for a comprehensive overview [2–5].

In agent-based cancer models, each agent represents a single tumour cell or cell cluster. In this paper, we settle for a lattice-based cellular automata (CA) model [6, 7]. This choice implies that each tumour cell is associated with a lattice site. For simplicity, we confine our tumour cells to a two-dimensional plane — as in the case of cell growth *in vitro*, i.e. in a cell-culture dish. To avoid artefacts stemming from the geometry of the lattice, we use an irregular grid (cf. Voronoi tessellation [8]). A Cartesian lattice would thus lead to a square tumour, reflecting the symmetries of the lattice [1, 8, 9]. Our lattice contains 40,000 lattice sites and does not allow for cells or nutrients to flow beyond its outer boundary — cells at the boundary have no lattice sites to expand into, and we impose zero-flux (Neumann) boundary conditions on the nutrient flow.

Each cell is governed by a set of deterministic and stochastic rules to encode the dynamics of the cell population. By modelling the evolution of the system in discrete time steps, we successively apply these rules to each cell. For each time step, we randomly shuffle the order by which we cycle through the cells to avoid introducing spatial preferences and artefacts in the tumour growth.

At any given time step, each cell might migrate to a neighbouring lattice site, divide, or die. At any given time step, cells might die with probability P_{cd}. A cell can migrate with a probability P_{mig} if there is a free neighbouring lattice site to move to. Moreover, cells are not allowed to migrate if cell division takes place during the same time step (cf. the "Go or Grow" hypothesis for glioma, [10]). Since cell division leads to the creation of new cells, cell division can only happen if there is a lattice site available for the daughter cell [9]. If a cell is placed at the tumour edge, it might divide with probability P_{div}. Cells embedded within the tumour can also divide but must push their neighbours aside to do so. Since this effort costs energy, the probability of cell division decreases exponentially with the distance to the tumour edge [11, 12]. Here, we set the probability for cell division to zero if the next available spot is more than 8 lattice sites away. Only cells close to the edge, i.e. in the proliferating rim, are hence able to divide. The remaining bulk of the tumour is quiescent. This behaviour is consistent with the well-known growth patterns of spherical avascular tumours [13] (see also S1 Fig).

In the quiescent regions of the tumour, it is superfluous to spatially resolve individual cells since they can neither propagate nor proliferate. While we spatially resolve individual cells in the proliferating rim, we thus merge cells into clusters when they are situated deep within the tumour. In other words, to lower the computational cost, we employ an *adaptive* irregular lattice. For the clusters, we still monitor the individual cell fates, i.e. cell death.

Glioblastoma multiforme is hierarchically organised, which implies that the tumour is composed of different cell types [14]. This notion is important since each cell type is governed by different rules involving different probabilities (P_{mig}, P_{div}, and P_{cd}). We must, therefore, distinguish between glioblastoma stem-like cells (GSC), propagating progeny (GPP), and cells in a differentiating subpopulation (GDS). There are two possible outcomes when a GSC undergoes cell division: It either splits into two GSCs

with probability 1-ϵ or a GSC and a GPP with probability ϵ. A GPP, on the other hand, might divide into two GPPs or produce one GDS with equal probability. GDSs do not divide.

The cells inhabit an environment that contains nutrients. Initially, these nutrients are evenly distributed across the lattice. However, we have placed a nutrient source in the lower left-hand corner of the lattice, and the nutrients are locally consumed by the cells as the tumour grows. Gradients in the nutrient concentration (n) thus arise over time. The resulting diffusion of nutrients across the lattice is modelled using a reaction-diffusion equation on the form [15–17]

$$\frac{\mathrm{d}n}{\mathrm{d}t} = D\nabla^2 n - \lambda_\mathrm{C}\rho + S. \tag{1}$$

Here, D denotes the diffusion coefficient for the nutrient flow, λ_C scales the nutrient consumption of the cells, and ρ denotes the tumour cell concentration, i.e. we assume that all cell types consume the same. Finally, S signifies any nutrient sources, i.e. the aforementioned inflow of nutrients in the lower left-hand corner. At each time step, we solve Equation (1) using the Python Package FiPy [18]. Note that Equation (1) is solved on a Cartesian grid. At each time step, interpolation is required between this grid and the irregular lattice on which the cells live. We illustrate the described set-up in S2 Fig.

We assume that a sufficiently low nutrient concentration will inflict immediate cell death [19]. Such low concentrations might occur at the centre of the tumour leading to a necrotic core. This morphology is observed for real tumours.

For glioblastoma multiforme, the migration of tumour cells is known to primarily occur along white matter tracks, and blood vessels [20–22]. One might take this behaviour into account by letting the stochastic rules that govern cell actions vary across the lattice. In addition, one could allow for mutations that alter these rules. However, for our purposes, simple rules for the cell dynamics suffice. Our primary focus lies in extracting general guidelines for stochastic models rather than capturing all aspects of this specific brain cancer type.

Parameters and summary statistics

We initialise each simulation with a single GSC near the centre of the lattice and vary four input parameters ($\boldsymbol{\Theta}$):

- Our first parameter is the probability, P_div, for GSCs with a free neighbouring lattice site to divide during each time step. While GPPs are assumed to divide 0.6 times this rate, GDSs do not divide. Note that these rules are chosen for their simplicity rather than given a biological justification.
- The second parameter is the probability, P_cd, for GPPs to undergo programmed cell death during each time step. While GDSs are assumed to be three times more likely to die at every time step, GSCs do not die spontaneously. Again, these rules are chosen for their simplicity. Note that cells die irrespective of their cell type if the nutrient concentration falls below a critical level.
- Our third parameter is λ_C in Equation (1).
- Finally, we include ϵ that denotes the probability for GSCs to differentiate into GPPs during cell division.

Note that λ_C is the only parameter that is not a probability. However, we choose the values for the remaining parameters (D and S) in Equation (1) such that interesting scenarios arise when λ_C is varied between 0.0 and 1.0. While this choice is not

biologically motivated, it means that all parameters can be treated in the same way, which provides an interesting contrast to the epidemiological SIRDS model presented in the section *Epidemic, SIRDS model*.

We note that our cancer CA model has many more parameters than the ones we vary in our study. Among others, these parameters include D, S, and the critical nutrient level below which immediate cell death due to starvation occurs. We set these to 5 units of area per time step, 500 per time step at the lower-left corner, and 0.1 per unit area, respectively. We also do not vary the migration probability of the different cell types. For GSCs and GDSs, we set the probability for cell migration to 0.02, while GPPs migrate with a probability of 0.5. Due to the "Go or Grow" hypothesis, however, the migration probability of any individual cell is temporarily set to zero if the cell has undergone cell division within the same time step.

To decide on the summary statistics, we must consider the type of data that one might encounter in a cancer study. In an animal-based experiment, we might sacrifice the animal to study its brain. Alternatively, we might deal with magnetic resonance imaging (MRI) or in vivo imaging system (IVIS) data. In any case, we would know the time since the injection of the tumour cells into the animal. We hence assume that our data will comprise a detailed snapshot of the tumour rather than a time series for tumour growth. In the best-case scenario, we might have access to spatial transcriptomics (ST) data that enables us to distinguish different cell types and resolve single cells.

The raw cell count already provides extensive information about $\mathbf{\Theta}$. The reason for this is twofold. First, since we do not vary the initial conditions, variations in $\mathbf{\Theta}$ do not imply significant spatial variation that might affect tumour growth. Secondly, we do not intend to infer spatial properties, such as varying initial conditions or the impact of cell migration. Based on these considerations, we settle for the following five summary statistics:

- The number of alive glioblastoma stem-like cells (GSC) at $t = 100$.
- The number of alive propagating glioblastoma cells (GPP) at $t = 100$.
- The number of alive differentiating glioblastoma cells (GDS) at $t = 100$.
- The number of dead tumour cells at $t = 100$.
- The total number of tumour cells in the proliferating rim at $t = 100$.

As discussed above, we define the proliferating rim as those cells that are placed within 8 lattice sites of a free lattice site. Note that the maximal width of the proliferating rim (d_{rim}), i.e. here 8 lattice sites, is yet another parameter that we keep fixed in our analysis. Also, note that the probability for cell division is set to decrease for cells that are embedded within the proliferating rim, reflecting the work needed to push other tumour cells aside. For a tumour cell with distance d to the nearest free lattice site, the probability for cell division is thus set to be a factor of $\exp(-\frac{d}{2d_{\text{rim}}})$ smaller for $d < d_{\text{rim}}$.

Epidemic, SIRDS model

Agent-based models are in widespread use within the field of mathematical epidemiology. They have thus been applied to a broad variety of data, including studies of the SARS-CoV-2 pandemic [23, 24].

The considered agent-based models for infectious diseases fall into the category of compartmental epidemiological models [25]. Such models, at the very least, distinguish between the infected (I) and susceptible (S) individuals or subpopulations. In addition,

many diseases lead to immunity, and some infections might be fatal, making it essential to track recovered (R) individuals as well as the death toll (D). To indicate that our model takes these four categories into account, we refer to it as a SIRDS model. By repeating the reference to the susceptible individuals (S), we express that recovered individuals might become susceptible to the disease again after a certain period of immunity.

One might model the agents as a set of Brownian particles that move in a two-dimensional plane. Much can already be learned from such an approach, and the correct macroscopic behaviour can be reproduced [26]. This being said, human interactions are governed by rules that reflect socio-demographic structures. In addition to random encounters, people thus tend to have predictable encounters within a certain social group. Regular social interactions with the same individuals take place within households, at educational facilities or in workplaces. Agent-based models present an opportunity to include such social networks [24, 27–30].

In this paper, we consider agents on a square lattice (see [31–35] for other examples of lattice-based models). In addition to having $N_{\rm re}$ random encounters, each agent interacts with the agents at the eight neighbouring lattice points on a daily basis. Leaving random encounters aside, we note that the agents do not move throughout the simulation, i.e. the social networks are rigid. The distance between agents thus expresses social relationships rather than geographical proximity. In total, there are 12,100 (110×110) available lattice sites. Only 10,000 randomly selected sites are occupied to account for the variability of social groups. A susceptible agent that encounters an infected agent will be infected with a probability $P_{\rm i}$ (for each day of exposure). There are initially three infected individuals randomly scattered across the lattice. An infected individual will recover or die after a number of days drawn from an exponential distribution with mean $t_{\rm i}$. The agent dies with the probability $P_{\rm d}$. If the agent recovers, it will be protected from being infected during a number of days drawn from an exponential distribution with mean $t_{\rm p}$. The agent will then re-enter the population of susceptible individuals (see also S3 Fig).

We stress that our epidemiological SIRDS model is rather simplistic. For instance, factors such as an individual's socioeconomic group, sex, or age might play a role in mortality and infection rates. Many mathematical models thus build on census data to draw a more realistic picture [36, 37]. We do not take such information into account. However, the considered level of complexity is sufficient for our purposes. Rather than modelling a specific disease, our focus lies on the stochasticity that all epidemiological agent-based models share, irrespective of their level of detail.

Parameters and summary statistics

In the case of our SIRDS model, we vary five input parameters ($\boldsymbol{\Theta}$):

- The first parameter is the infection probability, $P_{\rm i}$, per day during contact with an infected individual. Since $P_{\rm i}$ denotes a probability, this parameter can take values between 0.0 and 1.0.

- Our second parameter is the mortality rate, $P_{\rm d}$, of the disease. Again, this parameter can take values between 0.0 and 1.0.

- As a third parameter, we include the time, $t_{\rm i}$, during which the infected person can transmit the disease. We evolve the system in time steps, $\mathrm{d}t$, of 0.1 days with an agent becoming contagious from the time step that follows the infection. As regards the model grids presented in the section *Model grid* in our paper, we vary this parameter on a linear scale from 1.0 to 30.0 days.

- Our fourth parameter is the time, t_{p}, during which a recovered individual is protected from getting the disease. We vary this parameter on a logarithmic scale between -2.0 and 4.0 when constructing the grids presented in the section *Model grid* in our paper. At low values for t_{p}, the individual does not build up any immunity. Such models are appropriate for some bacterial infections, including meningitis. At high values for t_{p}, the disease effectively confers lifelong immunity as in the case of some viral diseases, such as measles [38].

- Finally, we vary the number, N_{re}, of random encounters with the general population. For the grids in the section *Model grid* in our paper, we use a logarithmic scale from -2.0 to 2.0. The model is hereby, for instance, able to account for social distancing and lockdown measures.

In contrast to the brain cancer model, we hence include parameters on different scales.

Rather than being interested in the spatial properties of our specific model, we aim to gain insights into the general properties of agent-based models for infectious diseases. When choosing our summary statistics, we hence turn to the emergent properties of the simulations. These properties comprise the time evolution of the different subpopulations, i.e. $S(t)$, $I(t)$, $R(t)$, and $D(t)$. To facilitate an easy comparison with our cancer model, we distil these time series into five summary statistics:

- Our first measure is the duration of the outbreak. Note that the computation is interrupted if the duration exceeds 1825 days.

- Secondly, we include the total death toll at the end of the outbreak.

- The third measure is the largest number of infected individuals during the outbreak. Note that we only record the largest peak, although the epidemic might lead to several waves.

- Fourthly, we include the time at which the largest peak in infection numbers occurs. To motivate this choice, let's assume that the first peak in infection numbers is the largest. The ratio between the time at which it occurs and the total duration of the epidemic would then hint at the existence of multiple waves and help to constrain t_{p}.

- Finally, we include the largest number of recovered agents during the epidemic. In contrast, the number of recovered people at the end of the epidemic is not a suitable measure because we include scenarios where the disease does not confer immunity.

The Schlögl Model

The Schögl model [39–41] describes a chemical reaction network containing three species, X, A and B. The following reactions couple these species:

$$\begin{aligned} A + 2X &\xrightarrow{k_1} 3X \\ 3X &\xrightarrow{k_2} A + 2X \\ X &\xrightarrow{k_3} B \\ B &\xrightarrow{k_4} X \end{aligned}$$

We are dealing with (discrete state, continuous-time) stochastic processes. In contrast to our brain tumour and epidemic models, the Schögl model is neither spatially resolved

nor does it keep track of individual agents to describe the chemical reaction system. The parameters $k_1 - k_4$ are associated with the propensity functions, i.e. the probability that any of the four reactions take place during a time step given the concentration of each of the four species. We can compute the time evolution of the system using the Gillespie algorithm. In short, this algorithm uses a Monte Carlo method to compute the time until the next reaction. It then updates the system by randomly choosing one of the reactions based on the propensity functions. For more details, we refer the reader to Ilie et al. (2019) [41].

The advantage of stochastic over deterministic modelling of reaction networks lies in the fact that it correctly captures the probability distributions of the different species, wherever the number of participating molecules is finite. For instance, for some combinations of $k_1 - k_4$, the Schögl model famously exhibits bi-stability. As discussed below, however, bi-stabilities did not occur in any of our synthetic data.

To study the Schögl model, we constructed a grid containing 10,000 models varying $k_1 - k_4$ between 10^{-8} and 10^{-1}, sampling from the parameter space in a quasi-random manner. We initialised all systems with X, A and B at 100, 5,000 and 5,000 molecules, respectively. The computation of each reaction network was terminated after 50,000 reactions took place, which means that the final state of the system varies in four variables across the grid: the threes species (X, A, B) and the time T, at which the last computed reaction took place. Here, we do hence not assume that the populations of A and B are constant as it is commonly done.

We used the grid to train two emulators: a neural network (NN) and Gaussian Processes (GP). We then tested the performance of these emulators by using them to predict the output of 250 previously unseen sets of parameter values. For comparison, we ran the simulations 250 times for each of these 250 parameter sets to compute the real distributions of X, A, B, and T to which to compare. The results are summarised in S4 Fig.We note that bi-stabilities do not occur in our synthetic data sets, i.e. that the distributions of the different chemical species are seemingly uni-modal for all the 250 investigated quasi-randomly drawn parameter sets that constitute our synthetic data.

We find that both emulators predict the correct median of the variables (X, A, B, and T) with high accuracy. For nearly all the 250 test cases, the median is thus predicted with an accuracy that is better than an order of magnitude. However, in this case study, both emulators overestimate the stochastic nature of the chemical networks, which would affect any subsequent inference scheme. Once again, the NN outperforms the simple GP, even though both emulators predict the median similarly well and thus would score similarly well on many classical performance metrics, such as the coefficient of determination (R^2). This underlines one of our main statements: It is essential to explore and potentially discard several machine learning methods and metrics to tailor the emulation to the individual scientific case study.

In addition, we have trained two direct inference machines based on the grid of simulations: a neural network and Gaussian processes. We find that both direct inference machines can recover k_1 and k_2 with high accuracy. The NN yields much narrower posterior distributions than the GP, whereby it outperforms the GP in terms of $-\log_{10}(q)$. We summarise these results in S5 Fig.

However, as regards k_3 and k_4, both the NN and GP systematically yield parameter estimates that lie close to the centre of the corresponding parameter intervals irrespective of the synthetic data. Picking a value close to the centre of the interval is a good choice if one were to guess blindly. At first glance, both approaches do hence not do a good job pinning down k_3 and k_4, which is reflected in our performance metrics in S5 Fig.

It is useful to take a look at how the different variables behave across the parameter space to understand why the direct inference machines perform better for k_1 and k_2

than for k_3 and k_4 We thus summarise the behaviour of X and T in S6 Fig. As can be seen from the figure, some clear patterns occur in the plane spanned by k_1 and k_2, suggesting that the corresponding reactions dominate the network. However, there seems to be little information about X and T in the planes spanned by k_3 and k_4. Likewise, the contour plots of A and B are noisy in the (k_3, k_4)-plane. Correspondingly, measurements on X, A, B and T reveal little about these two parameters. Thus, it is expected that k_3 and k_4 cannot be inferred with high accuracy and precision. Taking this into account, we note that the direct inference machines actually do very well for all four parameters at a low computational cost.

References

1. Liedekerke P, Palm M, Jagiella N, Drasdo D. Simulating tissue mechanics with agent-based models: Concepts, perspectives and some novel results. Computational Particle Mechanics. 2015;2. doi:10.1007/s40571-015-0082-3.

2. Wang Z, Butner JD, Kerketta R, Cristini V, Deisboeck TS. Simulating cancer growth with multiscale agent-based modeling. Seminars in cancer biology. 2015;30:70—78. doi:10.1016/j.semcancer.2014.04.001.

3. Harris L, Beik S, Murobushi Ozawa PM, Jimenez L, Weaver A. Modeling heterogeneous tumor growth dynamics and cell-cell interactions at single-cell and cell-population resolution. Current Opinion in Systems Biology. 2019;17. doi:10.1016/j.coisb.2019.09.005.

4. Metzcar J, Wang Y, Heiland R, Macklin P. A Review of Cell-Based Computational Modeling in Cancer Biology. JCO clinical cancer informatics. 2019;3:1—13. doi:10.1200/cci.18.00069.

5. Weerasinghe WMH, Burrage P, Burrage K, Nicolau D. Mathematical Models of Cancer Cell Plasticity. Journal of Oncology. 2019;2019:1–14. doi:10.1155/2019/2403483.

6. Radszuweit M, Block M, Hengstler J, Schöll E, Drasdo D. Comparing the growth kinetics of cell populations in two and three dimensions. Phys Rev E. 2009;79:051907. doi:10.1103/PhysRevE.79.051907.

7. Poleszczuk J, Macklin P, Enderling H. In: Agent-Based Modeling of Cancer Stem Cell Driven Solid Tumor Growth. vol. 1516; 2016.

8. Block M, Schöll E, Drasdo D. Classifying the Expansion Kinetics and Critical Surface Dynamics of Growing Cell Populations. Physical review letters. 2008;99:248101. doi:10.1103/PhysRevLett.99.248101.

9. Drasdo D. Coarse Graining in Simulated Cell Populations. Advances in Complex Systems (ACS). 2005;08:319–363. doi:10.1142/S0219525905000440.

10. Giese A, Bjerkvig R, Berens M, Westphal M. Giese A, Bjerkvig R, Berens ME, Westphal M.Cost of migration: invasion of malignant gliomas and implications for treatment. J Clin Oncol 21: 1624-1636. Journal of clinical oncology : official journal of the American Society of Clinical Oncology. 2003;21:1624–36. doi:10.1200/JCO.2003.05.063.

11. Jagiella N, Müller B, Müller M, Vignon-Clementel IE, Drasdo D. Inferring Growth Control Mechanisms in Growing Multi-cellular Spheroids of NSCLC Cells from Spatial-Temporal Image Data. PLoS computational biology. 2016;12(2):e1004412. doi:10.1371/journal.pcbi.1004412.

12. Jagiella N, Rickert D, Theis FJ, Hasenauer J. Parallelization and High-Performance Computing Enables Automated Statistical Inference of Multi-scale Models. Cell systems. 2017;4(2):194—206.e9. doi:10.1016/j.cels.2016.12.002.

13. Hoehme S, Drasdo D. Biomechanical and Nutrient Controls in the Growth of Mammalian Cell Populations. Mathematical Population Studies. 2010;17:166–187. doi:10.1080/08898480.2010.491032.

14. Lan X, Jörg DJ, Cavalli FMG, Richards LM, Nguyen LV, Vanner RJ, et al. Fate mapping of human glioblastoma reveals an invariant stem cell hierarchy. Nature. 2017;549(7671):227—232. doi:10.1038/nature23666.

15. Cristini V, Lowengrub J, Nie Q. Nonlinear simulation of tumor growth. Journal of mathematical biology. 2003;46(3):191—224. doi:10.1007/s00285-002-0174-6.

16. Zheng X, Wise S, Cristini V. Nonlinear simulation of tumor necrosis, neo-vascularization and tissue invasion via an adaptive finite-element/level-set method. Bulletin of mathematical biology. 2005;67(2):211—259. doi:10.1016/j.bulm.2004.08.001.

17. Lowengrub J, Frieboes H, Jin F, Chuang YL, Li X, Macklin P, et al. Nonlinear modelling of cancer: bridging the gap between cells and tumours. Nonlinearity. 2010;23(1):R1—R9. doi:10.1088/0951-7715/23/1/r01.

18. Guyer JE, Wheeler D, Warren JA. FiPy: Partial Differential Equations with Python. Computing in Science Engineering. 2009;11(3):6–15. doi:10.1109/MCSE.2009.52.

19. Mansury Y, Kimura M, Lobo J, Deisboeck TS. Emerging patterns in tumor systems: simulating the dynamics of multicellular clusters with an agent-based spatial agglomeration model. Journal of theoretical biology. 2002;219(3):343—370. doi:10.1006/jtbi.2002.3131.

20. Giese A, Westphal M. Glioma invasion in the central nervous system. Neurosurgery. 1996;39(2):235—50; discussion 250—2. doi:10.1097/00006123-199608000-00001.

21. Swanson K, Alvord E, Murray J. A quantitative model for differential motility of gliomas in grey and white matter. Cell proliferation. 2000;33(5):317—329. doi:10.1046/j.1365-2184.2000.00177.x.

22. Hillen T, Painter KJ. In: Lewis MA, Maini PK, Petrovskii SV, editors. Transport and Anisotropic Diffusion Models for Movement in Oriented Habitats. Berlin, Heidelberg: Springer Berlin Heidelberg; 2013. p. 177–222. Available from: https://doi.org/10.1007/978-3-642-35497-7_7.

23. Hoertel N, Blachier M, Blanco C, Olfson M, Massetti M, Rico M, et al. A stochastic agent-based model of the SARS-CoV-2 epidemic in France. Nature Medicine. 2020;26:1–5. doi:10.1038/s41591-020-1001-6.

24. Silva P, Batista P, Seixas Lima H, Alves M, Pedrosa Silva R, Guimarães F. COVID-ABS: An agent-based model of COVID-19 epidemic to simulate health and economic effects of social distancing interventions. Chaos, Solitons & Fractals. 2020;139. doi:10.1016/j.chaos.2020.110088.

25. Tolles J, Luong T. Modeling Epidemics With Compartmental Models. JAMA. 2020;323. doi:10.1001/jama.2020.8420.

26. Norambuena A, Valencia FJ, Guzmán-Lastra F. Understanding contagion dynamics through microscopic processes in active Brownian particles. Scientific Reports. 2020;10. doi:10.1038/s41598-020-77860-y.

27. Ferguson N, Cummings D, Cauchemez S, Fraser C, Riley S, Meeyai A, et al. Strategies for Containing an Emerging Influenza Pandemic in Southeast Asia. Nature. 2005;437:209–214.

28. Khalil K, Abdelaziz M, Nazmy TT, Salem A. An agent-based modeling for pandemic influenza in Egypt; 2010. p. 1 – 7.

29. Ajelli M, Gonçalves B, Balcan D, Colizza V, Hu H, Ramasco JJ, et al. Comparing large-scale computational approaches to epidemic modeling: Agent-based versus structured metapopulation models. BMC infectious diseases. 2010;10:190. doi:10.1186/1471-2334-10-190.

30. Hoertel N, Blachier M, Blanco C, Olfson M, Massetti M, Limosin F, et al. Facing the COVID-19 epidemic in NYC: a stochastic agent-based model of various intervention strategies. medRxiv : the preprint server for health sciences. 2020;doi:10.1101/2020.04.23.20076885.

31. de Souza DR, Tomé T. Stochastic lattice gas model describing the dynamics of the SIRS epidemic process. Physica A Statistical Mechanics and its Applications. 2010;389(5):1142–1150. doi:10.1016/j.physa.2009.10.039.

32. Tomé T, Ziff RM. Critical behavior of the susceptible-infected-recovered model on a square lattice. Physical Review E. 2010;82(5):051921. doi:10.1103/PhysRevE.82.051921.

33. Taghvaei A, Georgiou T, Norton L, Tannenbaum A. Fractional SIR Epidemiological Models. medRxiv : the preprint server for health sciences. 2020;doi:10.1101/2020.04.28.20083865.

34. Mukhamadiarov R, Deng S, Serrao S, P, Nandi R, Yao L, et al. Social distancing and epidemic resurgence in agent-based susceptible-infectious-recovered models. Scientific Reports. 2021;11. doi:10.1038/s41598-020-80162-y.

35. Pont MT, Cortes J, Mora H. An Epidemic Grid Model to Address the Spread of Covid-19: A Comparison Between Italy, Germany and France. Mathematical and Computational Applications. 2021;26:14. doi:10.3390/mca26010014.

36. Hunter E, Namee BM, Kelleher JD. An open-data-driven agent-based model to simulate infectious disease outbreaks. PLoS ONE. 2018;13.

37. Hunter E, Kelleher JD. Adapting an Agent-Based Model of Infectious Disease Spread in an Irish County to COVID-19. Systems. 2021;9(2). doi:10.3390/systems9020041.

38. Hethcote HW. In: Levin SA, Hallam TG, Gross LJ, editors. Three Basic Epidemiological Models. Berlin, Heidelberg: Springer Berlin Heidelberg; 1989. p. 119–144. Available from: https://doi.org/10.1007/978-3-642-61317-3_5.

39. Schlögl F. Chemical reaction models for non-equilibrium phase transitions. Zeitschrift für Physik. 1972;253:147–161. doi:10.1007/BF01379769.

40. Rathinam M, Petzold L, Cao Y, Gillespie D. Consistency and Stability of Tau-Leaping Schemes for Chemical Reaction Systems. Society for Industrial and Applied Mathematics. 2005;4:867–895. doi:10.1137/040603206.

41. Ilie S, Enright WH, Jackson K. Numerical solution of stochastic models of biochemical kinetics. The Canadian Applied Mathematics Quarterly. 2009;17.
